# Reversible rituximab-induced rectal Kaposi’s sarcoma misdiagnosed as ulcerative colitis in a patient with HIV-negative follicular lymphoma

**DOI:** 10.1186/s13569-018-0097-7

**Published:** 2018-06-11

**Authors:** Emilien Billon, Anne-Marie Stoppa, Lena Mescam, Massimo Bocci, Audrey Monneur, Delphine Perrot, François Bertucci

**Affiliations:** 1 INSERM UMR1068, CNRS UMR725, Department of Medical Oncology, Centre de Recherche en Cancérologie de Marseille (CRCM), Institut Paoli-Calmettes, 232 Bd de Sainte-Marguerite, 13009 Marseille, France; 2 Department of Hematology, Institut Paoli-Calmettes, Marseille, France; 3 Department of Pathology, Institut Paoli-Calmettes, Marseille, France; 4 Department of Digestive Endoscopy Centre Hospitalier Edmond Garcin, Aubagne, France; 5 Faculty of Medicine, Aix-Marseille University, Marseille, France; 6 French Sarcoma Group, Paris, France

**Keywords:** Follicular lymphoma, Immune suppression, Kaposi sarcoma, Rituximab

## Abstract

**Background:**

Kaposi’s sarcoma is a low-grade mesenchymal angioproliferative tumor, most commonly observed in immunocompromised individuals, such as HIV-infected patients. Iatrogenic Kaposi’s sarcoma occurs in patients undergoing immunosuppressive therapies. Rituximab is a chimeric monoclonal antibody targeted against the pan B cell marker CD20. Because of its immunosuppressive effects through reduction of mature B-cells, it may exacerbate Kaposi’s sarcoma in HIV-positive patients. Rituximab-related Kaposi’s sarcomas have been previously reported in only two HIV-negative patients and were treated surgically.

**Case presentation:**

Here, we report on a Kaposi’s sarcoma that developed under rituximab treatment in a HIV-negative 55-year-old patient treated for follicular lymphoma. The lesion developed during the maintenance rituximab therapy at the rectal level with an aspect of apparent ulcerative colitis, without any cutaneous lesion. The premature stop of rituximab led to the complete regression of Kaposi’s sarcoma, without any additional specific treatment.

**Conclusions:**

To our knowledge, this is the third case of Kaposi’s sarcoma diagnosed under rituximab in a HIV-negative patient, the first one at the rectal level and the first one that completely regresses after stop of rituximab. This case raises awareness of iatrogenic Kaposi’s sarcoma in HIV-negative patients treated with rituximab, and further highlights the importance of immunosuppression in the pathophysiology of disease.

## Background

Kaposi’s sarcoma is a low-grade mesenchymal angioproliferative tumor caused by the lytic replication of human herpesvirus type 8 (HHV8), identified with Polymerase Chain Reaction (PCR) in 95% of cases. The lesions predominantly present at muco-cutaneous sites, but may involve all organs and anatomic locations. Kaposi’s sarcoma occurs most commonly in immunocompromised individuals such as HIV-infected patients. Iatrogenic Kaposi’s sarcoma occurs in patients undergoing immunosuppressive therapies for autoimmune disorders or after organ transplantation [1].

Rituximab is a chimeric murine/human monoclonal antibody (mAb) targeted against the pan B-cell marker CD20. It was the first mAb to receive approval by the Food and Drug Administration for use in cancer treatment. Since its approval for relapsed/refractory non-Hodgkin’s lymphoma in 1997, rituximab has been licensed for use in the treatment of numerous other B-cell malignancies, including the follicular lymphoma [2], as well as autoimmune conditions, including rheumatoid arthritis. Because of its immunosuppressive effects through action on CD20 and reduction of mature B-cells, rituximab therapy may exacerbate Kaposi’s sarcoma in HIV-positive patients [3]. Rituximab-related Kaposi’s sarcomas have been previously reported in two HIV-negative patients, without multicentric Castelman’s disease [4, 5], and were treated surgically.

We herein report a rectal Kaposi’s sarcoma that developed under rituximab treatment in a HIV-negative patient treated for follicular lymphoma, and that completely regressed upon cessation of rituximab, without any additional specific treatment.

## Case presentation

**Fig. 1:**
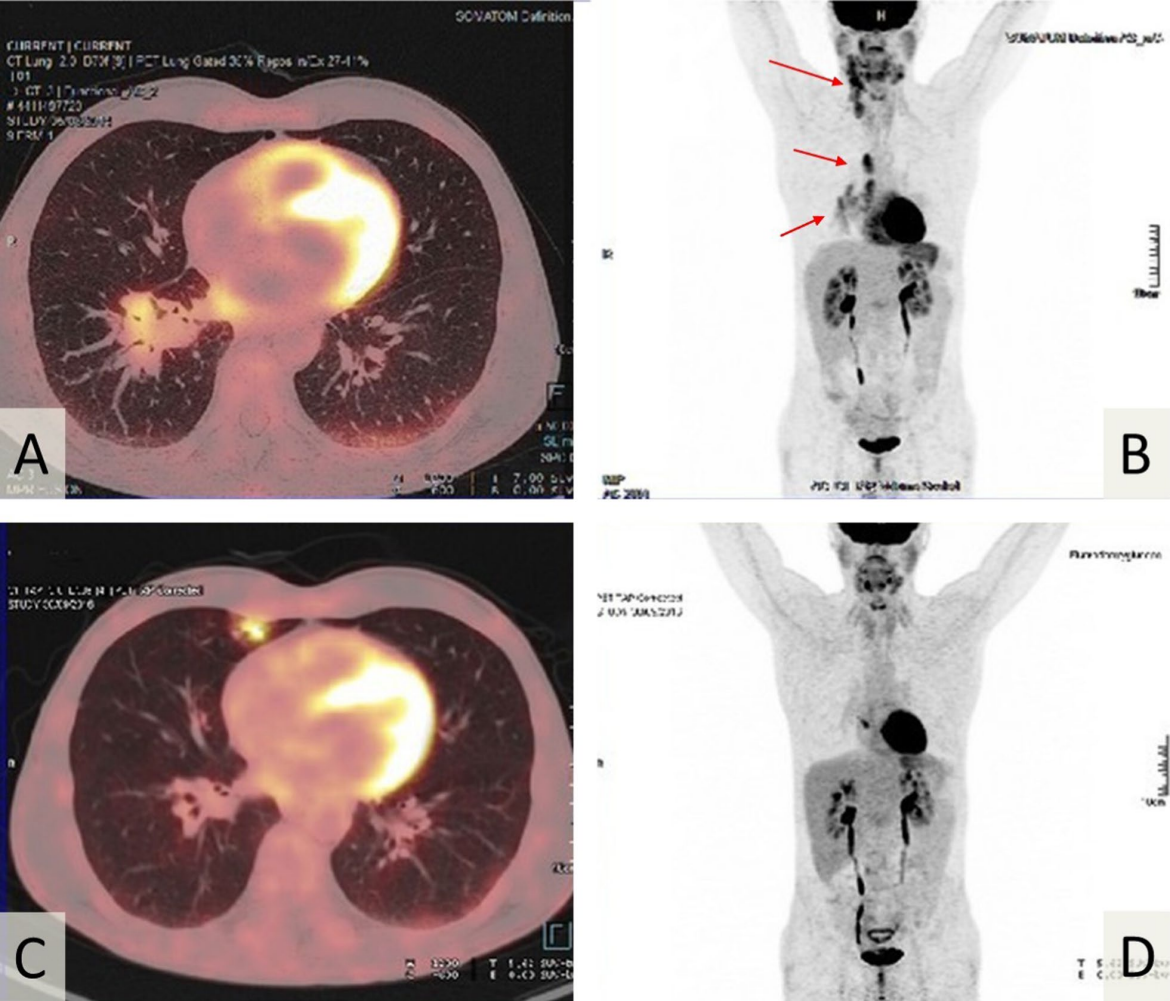
PET-scan imaging. **A**, **B** PET-scan of July 2014 at time of diagnosis, showing hypermetabolic cervical, mediastinal and hilar lymphadenopathies (red arrows). There was no digestive localization. **C**, **D** PET-scan of October 2016, showing pulmonary FDG uptake compatible with lung infection. No suspect FDG uptake of lymphoma recurrence or digestive uptake was found

**Fig. 2:**
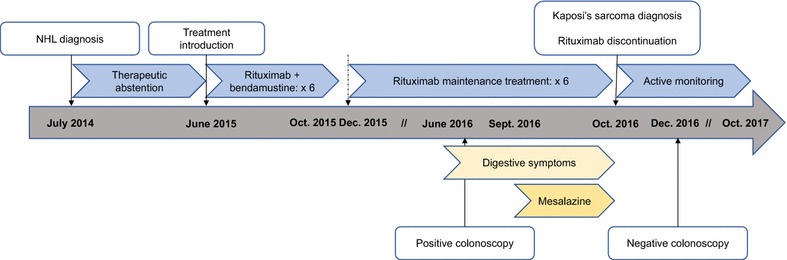
Timeline of patient’s care

In July 2014, a 55-year-old Caucasian man with cervical and mediastinal polyadenopathies was diagnosed with a non-Hodgkin follicular lymphoma (WHO grade 2) in our institution (Fig. 1A, B). There was no clinical general symptom, and the disease stage was II. He had no specific personal or familial medical history. Because of the low malignancy grade and the low tumor mass, no treatment was introduced, and a monitoring was set up. In June 2015, because of increase in size of cervical adenopathies, therapy combining rituximab (375 mg/m^2^) and bendamustine (90 mg/m^2^) was initiated. Six monthly cycles were delivered, with good tolerance and complete metabolic response (PET-scan of October 2015), then followed by maintenance rituximab (375 mg/m^2^, 1 injection every 2 months) started in December 2015 and planned for 2 years (Fig. 2) [6].

**Fig. 3:**
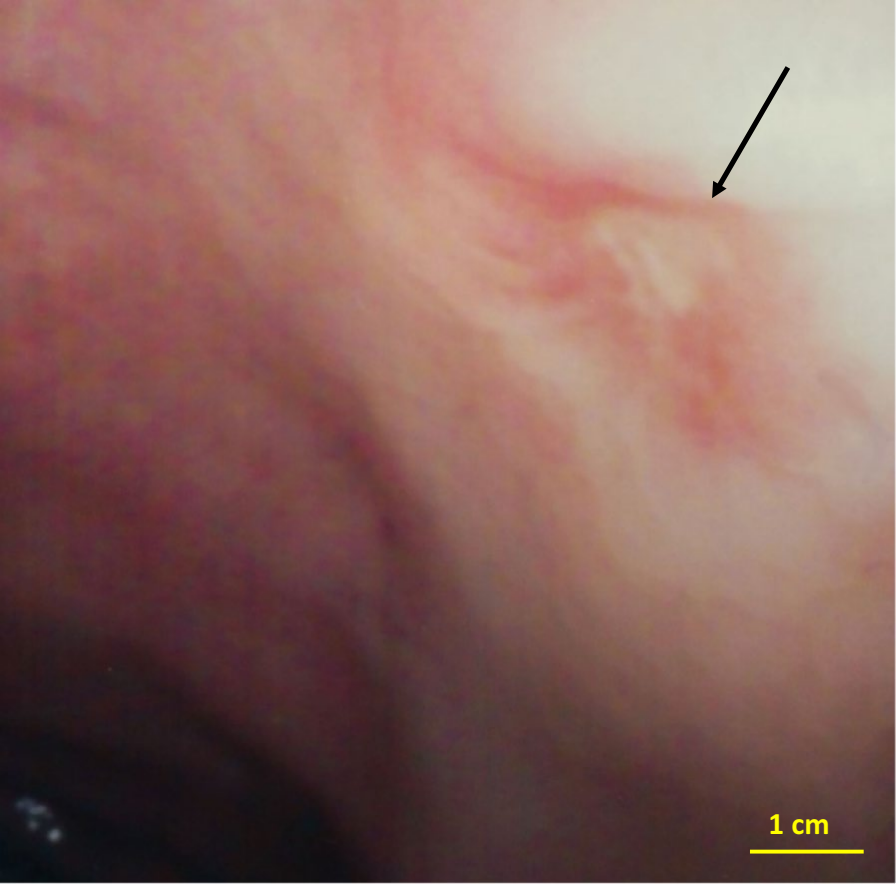
Colonoscopy imaging. Colonoscopy of June 2016 showing sigmoiditis, with a 4-mm-depth rectal ulceration (black arrow) at 20 cm of the anal canal, compatible with ulcerative colitis

**Fig. 4:**
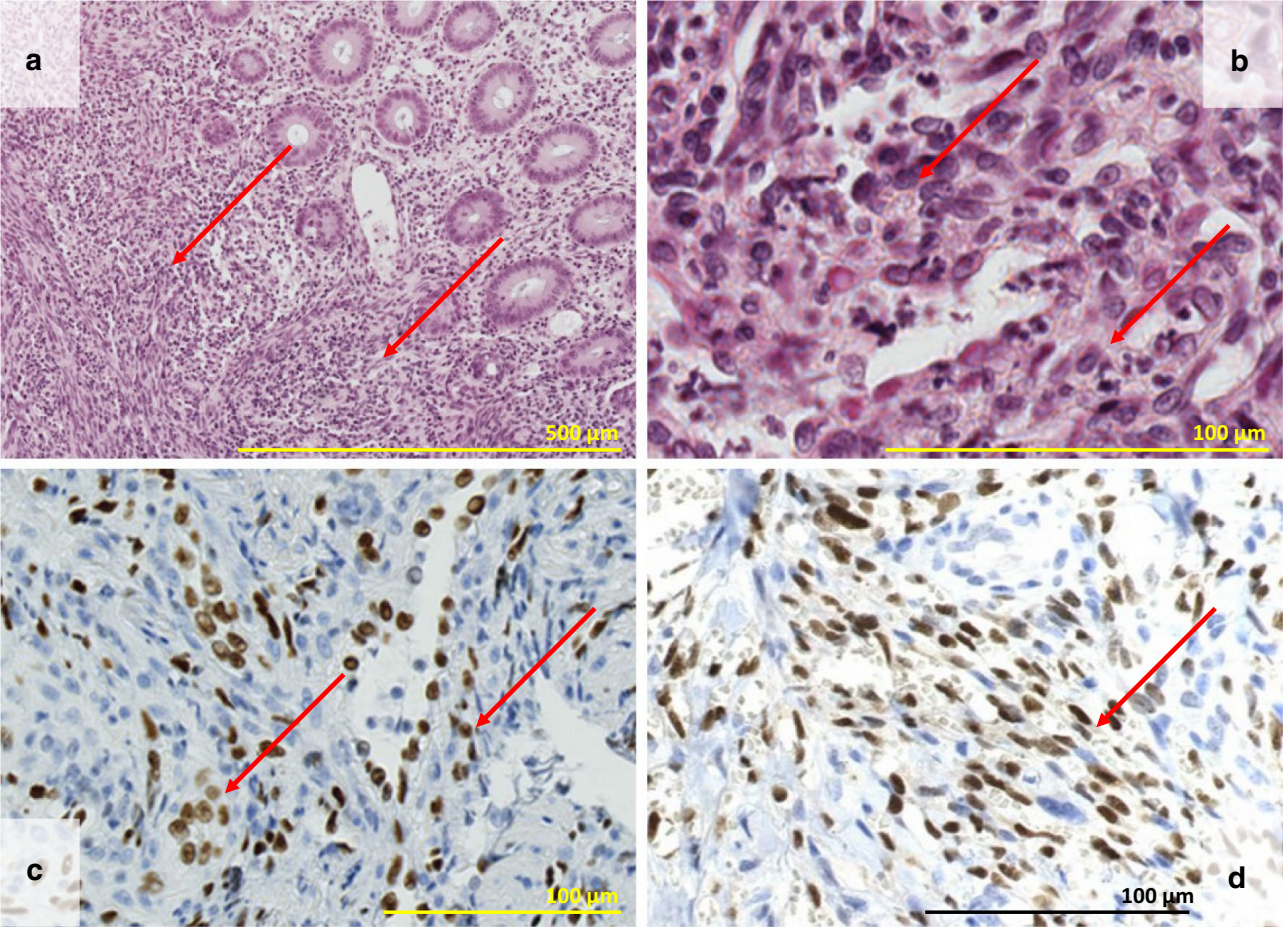
Pathological aspect of the rectal Kaposi’s sarcoma. **a** Microscopic aspect of the biopsy of rectal ulceration: the rectal mucosa is infiltrated by an ill-defined cellular fasciculated to diffuse proliferation (HES ×10). **b** Microscopic aspect showing spindle cells with little to moderate nuclear atypia surround vascular clefts. Few mitoses are noted. Lymphocytes and plasma cells are admixed (HES ×40). **c** IHC with ERG antibody: the lining cells of vascular structures and spindle cells express the ERG endothelial marker (×20). **d** IHC with HHV8 antibody: see the nuclear positive immunostaining of spindle tumor cells (×40)

In June 2016, before the fourth maintenance injection, the patient developed diarrhea alternating with constipation, nausea, associated with weight loss. Colonoscopy showed sigmoiditis, with a 4-mm-depth rectal ulceration at 20 cm of the anal canal (Fig. 3), compatible with ulcerative colitis. Multiple biopsies were performed, and the provisional diagnosis of ulcerative colitis was retained before availability of pathological results. No fecal calprotectin measurement was done. Mesalazine therapy (2 g/day) was introduced in September 2016. Pathological analysis of the colic biopsies showed aspects of non-specific subacute colitis. However, the rectal biopsy showed a spindle-cell proliferation with high cell density. Cell atypia were moderate, the cytoplasm was scarce, and the nuclei were slightly dyscaryotic, with rare mitoses. There was no necrosis. Immunohistochemistry (IHC) revealed positive staining of cancer cells for CD34 and negative for CD117. The diagnosis of Kaposi’s sarcoma was suspected, and the samples were sent to our Department of Pathology for reviewing by expert pathologist within the French Sarcoma Network (Réseau de Référence en Pathologie des Sarcomes, RRePS). The diagnosis of rectal Kaposi’s sarcoma was confirmed in October 2016: there was an ill-defined, fasciculated to diffuse proliferation of spindle cells with little to moderate nuclear atypia and few mitoses, outlining vascular slots; IHC showed positive staining of tumor cells for CD31 and ERG endothelial markers and for HHV8, and negative staining for CD117, DOG1, and STAT6 (Fig. 4). No cutaneous lesion was present. The patient stopped mesalazine after 1 month treatment because of relief of digestive symptoms and the diagnosis of ulcerative colitis was not finally retained because of pathological results Blood tests did not detect HHV-8 viremia, and the patient was serologically negative for HIV-1 and HIV-2, hepatitis B and C, and HTLV1 viruses. The circulating CD4+ T-cell count was 387/mm^3^.

Given the description in the literature of rituximab-induced Kaposi’s sarcoma in HIV-positive patients, rituximab was prematurely discontinued in October 2016 after the sixth maintenance injection. At this time, PET-scan showed neither suspect hypermetabolism of lymphomatous recurrence, nor digestive FDG uptake likely because of the disappearance of colitis after mesalazine treatment and the low proliferation rate of sarcoma. We noted only the appearance of lung lesions of infectious appearance without concomitant respiratory and infectious clinical symptoms (Fig. 1C, D). In December 2016, after complete disappearance of digestive symptoms, the patient underwent a colonoscopy, which was strictly normal without any sign of rectal Kaposi’s sarcoma or colitis. Because of the infectious images on the PET-scan, explorations were carried out with pneumocystis PCR, and CMV, HSV, and HTLV1 serologies. All these explorations remained negative, as well as a bronchoscopy. Of note, in July 2017, the colonoscopy was normal, notably at the rectal level. In October 2017, 12 months after the last dose of rituximab maintenance, our patient was still in complete remission of his lymphoma.

## Discussion and conclusions

We report a case of rectal Kaposi’s sarcoma likely induced by rituximab therapy in a HIV-negative patient treated for a follicular lymphoma. The lesion developed while being treated with maintenance rituximab, leading to prematurely stop the treatment. Interestingly, stopping rituximab allowed the complete regression of Kaposi’s sarcoma. To our knowledge, this is the first description of such case in the literature.

Two other cases of rituximab-induced Kaposi’s sarcoma in HIV-negative patients have been reported in the literature. One case of cutaneous Kaposi’s sarcoma was described in a patient treated with rituximab for thrombotic thrombocytopenic purpura. The lesion was treated with cryodestruction [4]. Another case was reported in an 84-year-old patient after undergoing rituximab-containing chemotherapy (R-CHOP regimen) for the treatment of a diffuse large B-cell lymphoma (DLBCL); after the seventh cycle, the patient developed a severe bacterial pneumonia and subsequent CMV viremia. The cutaneous Kaposi’s sarcoma developed after the complete resolution of pneumonia and was treated with surgical resection [5]. Our case is the first rituximab-induced Kaposi’s sarcoma that developed at the mucosa level. The diagnosis of Kaposi’s sarcoma of the bowel was difficult to establish immediately in the absence of skin and oral lesions, and a provisional diagnosis of ulcerative colitis was made before availability of pathological results. We suppose that the inflammation present in the mucosa was secondary to the underlying submucosal Kaposi’s sarcoma ulceration. Kaposi’s sarcoma of the bowel presenting as apparent ulcerative colitis has already been reported in HIV-positive patients several years ago, when the pathological diagnosis used antibodies for IHC less sensitive and specific than now [7, 8]. But, intestinal Kaposi’s sarcoma has also been reported during the last decades in HIV-negative patients treated with immunosuppressive drugs for ulcerative colitis or Crohn disease, mimicking acute flare of colitis. Table 1 summarizes the cases reported since 2000 [9–22], as well as the other cases diagnosed in HIV-negative patients treated with immunosuppressive drugs after organ transplantation or for another inflammatory disease. In our case, we observed the complete resolution of Kaposi’s sarcoma 2 months after rituximab discontinuation without the use of any specific treatment. To our knowledge, this is the first case of reversible rituximab-induced KS described in the literature. In their review, Antman and Chang [23] reported several examples of Kaposi’s sarcoma regressions in renal transplant recipients after cessation, reduction or modification of immunosuppressive therapy. However, such modification led to graft rejection in approximately half of the patients. Similarly, in a HIV-negative patient treated with corticosteroid for idiopathic thrombocytopenic purpura, the skin Kaposi’ tumors regressed soon after discontinuation of corticosteroid therapy, and no recurrence was observed during the 30-month follow-up period [24]. In our case, we suppose that the rituximab cessation permitted immune restitution and regression of Kaposi’s sarcoma.

**Table 1:** Published cases of intestinal Kaposi’s sarcoma in HIV-negative patients after 2000

First author	Year	Sex	Age at diagnosis (years)	Clinical conditions	Visceral KS localizations	Immunosuppression therapy	Tumor HHV8 status	Blood HHV8 status	KS management
Cohen	2001	F	67	Crohn disease	Ileum	Prednisone	ND	ND	Discontinuation of immunosuppressive therapy
Kang	2004	F	60	Ulcerative colitis	Colon	Prednisone	+	ND	ND
Nepomniashchaia	2004	M	41	Allogenic kidney transplantation	Stomach, intestinal mesentery, cerebral	ND	ND	ND	ND
Bursics	2005	M	49	Ulcerative colitis	Colon	Methylprednisolone	−	−	Discontinuation of immunosuppressive therapy and coloprotectomy
Svrcek	2009	M	62	Ulcerative colitis	Rectum	Steroid, azathioprine	+	+	Discontinuation of immunosuppressive therapy and coloprotectomy
Girelli	2009	M	43	Ulcerative colitis	Descending colon	Prednisone, mesalazine, ciclosporin	+	−	Discontinuation of immunosuppressive therapy and coloprotectomy
Rodriguez	2010	M	65	Ulcerative colitis	Colon	Prednisone, methotrexate	+	+	Discontinuation of immunosuppressive therapy and coloprotectomy
Tas	2012	F	77	Chronic anemia	Colon, lymph nodes	None	+	ND	Etoposide
Pioche	2013	M	49	Ulcerative colitis	Colon	Corticosteroids, azathioprine, ciclosporin, infliximab	+	+	Coloprotectomy
Hamzaoui	2013	M	30	Ulcerative colitis	Colorectal	Prednisone, mesalazine, azathioprine, infliximab	+	ND	Subtotal colectomy
Herculano	2014	M	63	Ulcerative colitis	Sigmoid colon	Prednisolone, mesalazine	+	−	Discontinuation of immunosuppressive therapy
Windon	2017	M	21	Crohn disease	Colon	Prednisone, infliximab	−	−	Discontinuation of immunosuppressive therapy
Duh	2017	M	48	Ulcerative colitis	Rectum	Prednisone	+	ND	Discontinuation of immunosuppressive therapy followed by subtotal colectomy
Kumar	2017	M	70	Ulcerative colitis	Ascending colon	None	+	ND	ND

*KS* Kaposi’s sarcoma, *F* female, *M* male, *ND* not determined

The role of CD4 T-cells in the Kaposi’s sarcoma pathophysiology is suspected [25], especially in HIV-positive patients who usually present Kaposi’s sarcoma when circulating CD4+ T-cell count is under 350/mm^3^ under highly active antiretroviral therapy [26]. In our case as well as in Ureshino’s case, the CD4+ T-cell count was higher than 350/mm^3^ [5], suggesting that cellular immunodeficiency played only a minor role in the development of Kaposi’s sarcoma in these patients. As B-cells are the HHV8’s main human reservoir, another hypothesis may be that B-cell depletion induced by rituximab can expose endothelial cells to high HHV8 level, causing latent viral infection, and promoting Kaposi’s sarcoma development [23]. In HIV-positive patients, lower B-cell counts are associated with the risk of Kaposi’s sarcoma development [27], suggesting a role of humoral immune system in disease etiopathogenesis. One study, which investigated the pathological findings in a HIV-positive patient with rituximab-related Kaposi’s sarcoma who was treated for multicentric Castleman’s disease, showed depletion of intralesional B-lymphocytes accompanied with an upregulation of the HHV8 gene product K5 [28]. These authors also postulated that the diminished B-lymphocyte count interfered with the normal immune response to HHV8, allowing for viral activation. Unfortunately, the circulating CD20+ cell count and the Ig levels were not available for our patient, but they were low in the Ureshino’s case [5]. Despite the widespread use of rituximab, there has been only one case of rituximab-related Kaposi’s sarcoma in HIV-negative patients with DLBCL [5], and it was suggested that additional factors causing systemic inflammation, such as an infection, might contribute to the development of Kaposi’s sarcoma in addition to rituximab. In our patient, lung infection may have triggered Kaposi’s sarcoma progression.

In conclusion, we report the case of Kaposi’s sarcoma diagnosed under rituximab in a HIV-negative patient, the first one at the rectal level, and the first one that completely regressed after cessation of rituximab. This case further highlights the importance of immunosuppression in the pathophysiology of Kaposi’s sarcoma and how immune restitution takes part in its management. It also suggests awareness of iatrogenic Kaposi’s sarcoma in patients treated with rituximab. Even if the occurrence of Kaposi’s sarcoma is a very rare event, vigilance is needed, particularly for cancer patients often immunocompromised by disease and treatments. Patients at risk of HHV8 infection (ethnicity with high Fitzpatrick skin phototype, Mediterranean or equatorial African geographic origin, male gender, homosexuality or multiple sexual partners, immune deficiency) should be carefully screened for HHV8 before rituximab and closely monitored during treatment. Even if efforts to develop a HHV8 vaccine are ongoing [29], no prophylaxis for Kaposi’s sarcoma is available today. Finally, given the risk of lymphoma progression after rituximab cessation, further studies are needed to better understand the immunological mechanisms involved in rituximab-induced Kaposi’s sarcoma and to define optimal treatment.

## Abbreviations

CMV: cytomegalovirus
DLBCL: diffuse large B-cell lymphoma
HHV8: human herpes virus type 8
HIV: human immunodeficiency virus
HSV: herpes simplex virus
HTLV1: human T-cell lymphotropic virus de type I
IHC: immunohistochemistry
mAb: monoclonal antibody
PCR: polymerase chain reaction
PET-scan: positron emission tomography scan
RRePS: Réseau de Référence en Pathologie des Sarcomes
WHO: World Health Organization
